# Association between Dietary Patterns and All-Cause Mortality in the Chinese Old: Analysis of the Chinese Longitudinal Healthy Longevity Survey Cohort

**DOI:** 10.3390/nu16111605

**Published:** 2024-05-24

**Authors:** Yufei Chen, Ying Gao, Yexin Chen, Zuxin Wang, Huifang Xu, Fan Hu, Yong Cai

**Affiliations:** 1 School of Public Health, Shanghai Jiao Tong University School of Medicine, Shanghai 200025, China; chenyufly@sjtu.edu.cn (Y.C.); wangzuxin06@163.com (Z.W.); xuhuifang0812@163.com (H.X.); 2 Department of Public Health, Tongren Hospital, Shanghai Jiao Tong University School of Medicine, Shanghai 200050, China; yvonne.y.gao@outlook.com; 3 School of Management, Fudan University, Shanghai 200433, China; 23210690131@m.fudan.edu.cn

**Keywords:** dietary pattern, all-cause mortality, Chinese elderly, principal component analysis (PCA), prospective cohort

## Abstract

Diet is one of the most important ways to intervene and promote the health of older adults and reduce all-cause mortality. This study aimed to investigate the association between dietary patterns and all-cause mortality in the Chinese old. This study involved 11,958 subjects aged 65–116 years in the Chinese Longitudinal Healthy Longevity Survey (CLHLS) from 2008 to 2018. Dietary patterns were derived from principal component analysis (PCA) with varimax rotation. Four dietary patterns were derived: the ‘milk–egg–sugar pattern’, ‘carnivorous pattern’, ‘healthy pattern’, and ‘northeastern pattern’. Cox proportional hazard models were built for males and females separately to estimate the relationship between different dietary patterns and all-cause mortality. After adjusting for all covariates, the milk–egg–sugar pattern played a reverse role in mortality risk in males and females in different quartiles. In the carnivorous pattern, only males in the fourth quartile were observed to have a significantly reduced mortality risk (HR = 0.84 (95% CI: 0.77–0.93)). Both genders benefited from the healthy pattern, which consistently lowered mortality risk across all quartiles (males: HR = 0.87 (95% CI: 0.84–0.89); females: HR = 0.95 (95% CI: 0.92–0.97)). The northeastern pattern also showed an inverse association with all-cause mortality in males (HR = 0.94 (95% CI: 0.92–0.97)) and females (HR = 0.96 (95% CI: 0.93–0.98)). This study showed the association between dietary patterns and all-cause mortality in the Chinese old, which is significant for further quantitative studies.

## 1. Introduction

The world is currently experiencing a continuous and accelerating trend towards aging. In China, the population aged 65 and above surpassed 210 million in 2022 [1], and is projected to reach 380 million by the year 2050 [2]. However, as individuals age, there is often a decline in health, resulting in a reduction in the quality of life and an imposition of a significant burden on both families and society [3]. China’s outlined “Healthy China 2030” plan explicitly emphasizes the importance and urgency of achieving healthy and active aging [4]. Therefore, enhancing the health status of the elderly and realizing healthy aging have become top priorities in current research areas related to the elderly.

The health of the elderly is closely linked to their diets. The focus of nutritional epidemiology has shifted from the intake of individual nutrients or single foods to examining the combined effects of foods and nutrients, giving rise to the concept of dietary patterns. Dietary patterns refer to the quantity, proportion, type, or combination of different foods, beverages, and nutrients in a diet, as well as the habitual frequency of their consumption. Increasingly, research suggests that adopting a rational dietary pattern may improve the health status of the elderly, enhancing their quality of life and even reducing overall mortality risk. For instance, in elderly cohort studies from the United States [5], Sweden [6], and Italy [7], higher scores on the Mediterranean Diet (MED) or modified Mediterranean Diet (mMED) were associated with lower overall mortality risk. Studies focused on the Dietary Approaches to Stop Hypertension (DASH) diet found an inverse relationship between DASH scores and overall mortality rates in elderly men in the United States [5] and older individuals in Iran [8].

Chinese elderly individuals, especially those residing in rural areas and those of advanced age, commonly face nutritional risks, malnutrition, and incorrect perceptions of healthy dietary practices primarily due to objective physiological factors such as declining organ functions and reduced energy needs as well as intricate socio-psychologic influences [9,10,11]. Thus, addressing these issues through dietary recommendations is crucial for promoting the achievement of “healthy aging” in China. However, there is currently a research gap in understanding the association between dietary patterns of Chinese elderly individuals and overall mortality. Considering the physiological differences among diverse ethnic populations, further research and discussions specific to this demographic are necessary to identify dietary patterns that effectively mitigate the risk of overall mortality among the elderly in China.

Hence, this study aims to utilize data from the Chinese Longitudinal Healthy Longevity Survey (CLHLS), employing principal component analysis to extract dietary patterns and a Cox proportional hazards model to explore the research hypothesis that dietary patterns are associated with all-cause mortality in Chinese elder adults, thus providing valuable insights into identifying dietary strategies that may reduce the risk of overall mortality in the aging Chinese population.

## 2. Materials and Methods

### 2.1. Study Population

This study is based on data from the Chinese Longitudinal Healthy Longevity Survey (CLHLS), which is China’s first and largest nationwide, community-based, longitudinal prospective follow-up survey of the older adult population organized by the PKU Center for Healthy Aging and Development. Details of the survey have been described before [12]. All questionnaires were completed by professionally trained investigators at participants’ homes, and the quality and credibility of the data have been verified and widely accepted in previous studies [13].A multistage, stratified cluster sampling method was used to recruit participants at baseline (1998) from 23 Chinese provinces, covering approximately 85% of the total Chinese population. The CLHLS aimed to fill the data gaps for Chinese older adults (aged ≥ 65 years), especially the oldest (aged ≥ 85 years), and to explore the determinants of and the factors impacting healthy aging. Considering the dramatic improvements in the Chinese economy and living conditions in the last two decades, the current study chose CLHLS data from 2008 to 2018, including four waves that were more relevant to the recent dietary situation of the elderly. After sequentially excluding subjects who were lost to death or follow-up before 2018, had missing time of death information, were missing important data (including on diet, smoking, drinking, and exercise), had survival times of 0 years, and were aged less than 65 years old, a total of 11,958 participants were included in this study, including 5046 males and 6912 females.

All participants gave informed consent for inclusion before they took part in the study. The study was approved by the Biomedical Ethics Committee of Peking University (IRB00001052–24713074).

### 2.2. Dietary Assessment

The dietary information in the CLHLS that was used in this study consisted of the frequency of 13 food groups, including fresh fruits, fresh vegetables, meat, seafood, eggs, soybeans, salty vegetables, sugar, garlic, dairy, nuts, mushrooms or algae, and tea, and the amount of staple food. The frequency of eating was divided into four categories: every day or nearly every day, usually, sometimes, and seldom or not; the amount of staple food was divided into four levels: 0–4 taels, 4–6 taels, 6–8 taels, and ≥8 taels per day (1 tael equals to 50 g).

We used the principal component analysis method of exploratory factor analysis to capture dietary patterns. The principal components obtained using the correlation matrix method in the principal component analysis were first rotated orthogonally using the variance maximization method with the aim of being more conducive to explaining the maximum variation in the original data. Finally, a certain number of principal components was identified based on the eigenvalues (λ > 1), the scree plot of the eigenvalues, and the interpretability of the rotation factors. A factor loading (FL) ≥ 0.4 was considered as the characteristic food for that dietary pattern [14,15].

In addition, the amount of staple food and the frequency of consumption of each food were transformed into a standardized score (z-score) via a correlation matrix that was used for the calculation of each participant’s dietary pattern score for each food group, weighted by its factor loading. Higher dietary pattern scores represented the closer that participant’s diet was to that pattern and the converse, the further away from it. Each individual was then categorized into quartile groupings for each different dietary pattern based on their scores. Quartile 4 (Q4) represented the most closely adherent to the dietary pattern.

### 2.3. Outcome

The outcome was all-cause deaths that occurred between 1 January 2008 and 31 December 2018. The survival of the participants was confirmed at each follow-up wave, and specific information about deceased elderly participants, such as the time of death, was voluntarily provided by their close family members during the questionnaire interview. Survival time was calculated from baseline date to time of death or overall last follow-up time. Those who could not be contacted or whose relatives could not be contacted during the follow-up period were defined as missing.

### 2.4. Covariates

All covariates were measured at baseline survey and were included and classified according to previous studies and available cohort data. Demographic data (age, gender, residence, education level, marital status), lifestyle information (smoking, alcohol consumption, exercise status, sleep duration), and diseases were all collected through interviews and standardized questionnaires. Age was categorized into 65–85 years, 85–100 years, and ≥100 years for the young elderly, the elderly, and the oldest old; gender was categorized into male and female; residence was categorized into urban (combining ‘city’ and ‘town’ in the original questionnaire) and rural; education level was categorized into uneducated (0 years) and educated (>0 years); marital status was categorized into currently married and living with spouse or other (divorced, widowed, separated, or never married); smoking status was categorized into never smoked, former smoker, and current smoker. The same was done for alcohol consumption and exercise status; sleep duration was categorized into three levels: less than or equal to 6 h, 6–8 h, and more than 8 h; and disease was categorized into yes or no groups based on the self-reported presence or absence of hypertension, diabetes, cardiovascular disease, and cancer. Body Mass Index (BMI) was calculated from the height (cm) and weight (kg) recorded in the field measurement (BMI = weight/height^2^, kg/m^2^) and categorized into wasting (BMI < 18.5 kg/m^2^), normal (18.5–23.9 kg/m^2^), overweight (24–27.9 kg/m^2^), and obese (BMI ≥ 28 kg/m^2^) according to the Chinese BMI classification standard. Cognitive function was scored using the modified Mini-mental State Examination (MMSE), with a full score of 30; 27–30 was classified as normal, 21–26 as mild cognitive impairment, 10–20 as moderate cognitive impairment, and 0–9 as severe cognitive impairment.

### 2.5. Statistical Analysis

Baseline characteristics are presented in subgroups according to status in the last wave. All variables were categorical and expressed as numbers (frequency %). Age, education level, smoking, alcohol consumption, exercise status, sleep duration, MMSE, and BMI were ordinal variables. Differences between groups were derived from the chi-square test, with *p* < 0.05 considered statistically significant. The baseline characteristics of the elderly in the CLHLS, expressed according to quartiles of four dietary pattern scores, are presented in Table S1 of the Supplementary Materials. Characterization of the frequency of intake of 13 food groups, as well as the amount of staple food consumed by older adults in the CLHLS, grouped by final status, are listed in Table S2 of the Supplementary Materials.

The associations between the quartile groups of different dietary patterns and all-cause mortality are represented using hazard ratios (HRs) and 95% confidence intervals (CIs) obtained from Cox proportional hazards models. The results are presented separately for males and females. The proportional hazard assumption was examined using Schoenfeld residuals, and no violation was found in the main variables. In model 1, only age was included; model 2 was adjusted for demographic information (including age, residence, education level, and marital status), and model 3 included all confounding factors (including factors in model 2 as well as smoking status, alcohol consumption, exercise status, sleep duration, BMI, cognitive functioning status, and disease). Of these, covariates with less than 100 missing values (28 for the education level and 60 for sleep duration) were included directly in the model; covariates with more than 100 missing values (BMI, cognitive functioning status) were assigned to a separate stratification. Finally, the analysis was stratified according to age to build a full model and gender was included for sensitivity analysis. The results are presented in Supplementary Table S3. R 4.2.1 was used for statistical analysis, and a two-sided *p*-value < 0.05 was considered statistically significant.

## 3. Results

### 3.1. Baseline Characteristics

A total of 11,958 participants, including 5046 males (mean age 85.04 years) and 6912 females (mean age 90.11 years) from the CLHLS cohort from 2008 to 2018 were included in the study. The specific baseline characteristics are given in Table 1 below. Among them, significant differences were observed in baseline characteristics of gender, age, education level, marital status, smoking, alcohol assumption, exercise status, sleep duration, cognitive function, BMI, and diseases between those who ultimately survived and those who died.

### 3.2. Dietary Patterns

The details about dietary frequency are listed in Supplementary Table S2. The dietary data were tested for suitability for use in principal component analysis using the Bartlett sphere test (*p* < 0.001) and the Kaiser–Meyer–Olkin test (KMO = 0.745 > 0.7). According to the conditions mentioned in the methods, we identified four dietary patterns, which together explained 46.25% of the variance in the dietary variables. The food with absolute factor loads ≥ 0.4 is considered characteristic of the pattern. A positive factor load indicates that this type of food is positively correlated with the pattern, while a negative correlation is observed if the factor load is negative. As a result, these dietary patterns were named ‘milk–egg–sugar pattern’ (dairy, eggs, sugar, soybeans, mushroom or algae), ‘carnivorous pattern’ (meat, seafood), ‘healthy pattern’ (amount of staple foods, fresh fruit, nuts, fresh vegetable, algae), and ‘northeastern pattern’ (salty vegetables, garlic, tea). Details about the eigenvalues of different components can be found in Figure S1 of the Supplementary Materials and the factor loads for each food group in the four dietary patterns are presented in Table 2.

### 3.3. Associations between Dietary Patterns and All-Cause Mortality

During the 54,573 person-years of follow-up (10 years), a total of 9615 (80.41%) death outcomes occurred, including 3967 (78.62%) males and 5648 (81.71%) females. We generated a basic model, a demographic model, and a full model to detect the associations between different dietary patterns and all-cause mortality based on gender. Detailed results are presented in Table 3 below. As a result, we found some discrepancies between males and females. Female participants in the fourth quartile of the milk–egg–sugar pattern had an increased risk of all-cause mortality compared to others in the lower quartile (Q4: HR = 1.09 (95% CI: 1.01–1.18), *p* = 0.026) after adjusting for all covariates, while lower all-cause mortality in males was significantly associated with the second quartile of this pattern in the full model (Q2: HR = 0.91 (95% CI: 0.84–0.99), *p* = 0.045). The highest quartile of the carnivorous pattern was a significant protective factor in males, whether or not we corrected for confounding factors (Q4: HR = 0.84 (95% CI: 0.77–0.93), *p* < 0.001), but there was no similar finding in females. The results of the healthy pattern and the northeastern pattern were similar in males and females. The healthy pattern was shown to be associated with lower all-cause mortality both in males and females, regardless of quartile and correction factors (males: HR = 0.87 (95% CI: 0.84–0.89), *p*-trend < 0.001, females: HR = 0.95 (95% CI: 0.92–0.97), *p*-trend < 0.001). As for the northeastern pattern, it was also inversely associated with all-cause mortality in males and females, but the three higher quartiles were significant for males (HR = 0.94 (95% CI: 0.92–0.97), *p*-trend < 0.001), while the third and fourth quartiles were significant for females (Q3: HR = 0.89 (95% CI: 0.83–0.96), *p* = 0.002, Q4: HR = 0.89 (95% CI: 0.82–0.96), *p* = 0.003).

## 4. Discussion

In this study, we confirmed four dietary patterns using principal component analysis of data collected using a simple food frequency questionnaire (FFQ). The protective dietary patterns and the risk-associated dietary patterns in Chinese elderly males and females were determined, separately, during 10 years of follow-up. PCA is one of the most commonly used posteriori methods for deriving dietary patterns, and its feasibility, repeatability, and interpretability of results have been verified in previous studies [16,17,18]. For the results, the dietary patterns were named based on their characteristic foods: the milk–egg–sugar pattern was rich in dairy, eggs, sugar, soybeans, and mushroom or algae; the carnivorous pattern was characterized by high-frequency intake of meat and seafood; the healthy pattern means taking in enough staple foods and frequently eating a meal containing fresh fruits, nuts, fresh vegetables, and algae; and the northeastern pattern consisted of eating salty vegetables, garlic, and tea, which is quite similar to the dietary preferences of people in northeastern China.

### 4.1. Association and Differences between Four Dietary Patterns and Gender-Specific All-Cause Mortality

It was observed that the highest quartile of the milk–egg–sugar pattern played a role in increasing the risk of all-cause mortality in females and the second quartile was associated with lower all-cause mortality in males, which may be the result of a combination of the undesirable effects of sugar and the complex effects of milk and eggs. Multiple current studies have shown the risk of sugar [19,20]; however, the question of whether eggs and milk are bad for the Chinese elderly remains. There are two possible reasons for this. On the one hand, although dietary guidelines recommend reducing the consumption of whole-fat dairy products because of the inverse effect of saturated fats on blood lipids and cardiovascular disease, this evidence is mainly from high-income countries with high dairy intake. A large multinational prospective cohort study with participants from 21 countries across 5 continents, including China, supported that dairy consumption may benefit mortality and cardiovascular disease, especially in low-income and middle-income countries where dairy consumption is much lower than in North America and Europe [21]. For eggs, research on a wide range of egg intake (from <1/week to ≥10/week) and a large sample size of 102,136 participants found that the association between egg consumption and all-cause mortality observed in China was more likely to be a U-shaped relationship rather than the J-shaped or linear one observed in the US and other countries [22]. This comprehensive difference may be the reason why global studies involving multiple countries did not find significant associations [23]. On the other hand, compared to the lower quartile, the top quartile of this pattern was more likely to include urban-dwelling, obese, oldest old participants; this pattern seemed to represent a period of rapid improvement in urban living conditions and increased attention to the importance of eggs and milk, although sweets were still preferred. As we all know, obesity and aging are significant risk factors for mortality. More females preferred sugar compared to males, but there was no difference in egg and dairy consumption between them. The baseline characteristics that differed in quartiles of dietary patterns are presented in Supplementary Table S1.

The results of the carnivorous pattern are contrary to those of some previous studies [24,25,26,27,28], which demonstrated a benefit for Chinese older males in the full model. However, the health benefits of different diets to a particular individual or population depend both on the health value and quality of the food itself, and on the amount of nutrients required by that individual or group. There is no doubt that the high-quality protein, micronutrients, unsaturated fatty acids, and other nutrients in meat, seafood, and dairy are important for the health of the elderly and the population as a whole [29,30,31]; however, their ultimate health effects are closely correlated with the amount consumed and the needs of different individuals. A study on national associations between individual dietary factors and deaths correlated with specific cardiometabolic diseases in the United States showed that low omega-3 fats from seafood was one of the important reasons for diet-related cardiometabolic deaths [27]. Similarly, fish consumption has been shown to reduce cardiovascular disease and promote health in multiple populations [32,33,34]; however, this is also probably a U-shaped correlation [35,36]. Additionally, in terms of the frequency of meat and seafood intake, only 27.6% and 6.6% of people ate them almost daily, respectively, with more of the elderly (85–100 years, 43.7%) and the oldest old (≥100 years, 21.8%) participants in the study (84.81%) having a normal or low BMI. Only 22.97% of the elderly self-reported having the diseases mentioned in the methods. Generally, for seniors in good health, a higher intake of meat and seafood, within appropriate limits, may be helpful, although some quantitative dietary research is needed to confirm this; however, this effect was only pronounced in males in the study. 

Among the four dietary patterns, the plant-based healthy pattern was the most important protective factor among males and females (males: HR = 0.87 (95% CI: 0.84–0.89), *p*-trend < 0.001, females: HR = 0.95 (95% CI: 0.92–0.97), *p*-trend < 0.001). The conclusions about vegetables, fruits, nuts, mushrooms, and so on, similar to the former results, have been shown in different populations [37,38,39]. The quality of plant foods has been shown to influence the effect [40]. Subsequent studies should consider dividing vegetable types into root, stalk, and leafy vegetables, etc. The sensitivity analysis showed that the healthy pattern was associated with significantly lower mortality in the 65–85 years and 85–100 years old age groups, and only the third quartile presented the same association in the oldest old (≥100 years) year group. These differences between ages are also reflected in other dietary patterns; therefore, 100 years may serve as a cut-off point.

The northeastern pattern was also significantly protective in both males and females and in the full model of sensitivity analysis across all three age groups, with the strongest effects occurring in the third quartile. Of these, the benefits of tea and garlic have been confirmed in multinational populations and meta-analyses [41,42,43,44] of antihyperlipidemic, antihyperglycemic, and antihypertensive drugs, etc., [45,46]. In addition, two studies on the association between the Japanese diet and survival time or all-cause mortality in middle-aged and old people in Japan included salty vegetables, also called pickles, as a positive score and reached the same conclusion as we did [47,48]. This may be due to special substances such as probiotics and prebiotics produced during the fermentation of vegetables [49]; the high salt and high sodium content may be responsible for the significant effects observed in the third quartile.

### 4.2. Strengths and Limitations

First, there are few studies on the association between dietary patterns and all-cause mortality in Chinese elderly people, and the CLHLS, a large prospective cohort, is trustworthy because of its large sample size, wide coverage, and good representativeness. In addition, previous studies on dietary patterns in China and abroad usually utilized more detailed and complex methods of dietary investigation, such as the 3-day 24-h dietary review method and the food frequency questionnaire, but this is undoubtedly difficult for the elderly, especially the oldest old. This may be one of the reasons for the paucity of studies in this area. Therefore, this study attempted to conduct a rough dietary survey to extract dietary patterns by asking about the frequency of consumption of several major food groups in order to provide ideas and a basis for designing a simplified version of a dietary questionnaire for studying dietary patterns, and which is suitable for the elderly. We found that a healthy pattern was characterized by enough fresh fruits, nuts, fresh vegetables, and algae, and the amount of staple foods was significantly beneficial to reducing the risk of all-cause mortality, independently of either gender or age group, in the Chinese old. However, this study has several limitations: firstly, the rough dietary survey cannot provide accurate dietary information, and the conclusions and recommendations can only be limited to the frequency of consumption and cannot provide a basis for recommendation of intake quantity. Secondly, the questions about staple foods only focused on the amount without breaking down the types, yet the health effects of refined rice and whole grains are different. Thirdly, self-reporting of diet information and other lifestyle factors may be subject to recall bias, which is extremely difficult to avoid in population studies. Finally, dietary patterns cannot be invariable. However, due to the excessive number of missing values in the dietary data collected in each follow-up after baseline, it was impossible to establish a time series prediction model. Therefore, there is certainly a bias in using only the baseline dietary pattern as an independent variable.

## 5. Conclusions

In conclusion, dietary patterns play a vital role in the all-cause mortality of the Chinese old; the effect varies somewhat between gender and age groups. Future studies need to add quantitative questions to the simplified FFQ, both to clarify the dose–response relationship and to provide a basis for future quantitative dietary recommendations.

## Figures and Tables

**Table 1:** Baseline characteristics of participants in the CLHLS, according to the final status.

	Level	Total (N = 11,958)	Status		*p* *
			Alive (N = 2343)	Dead (N = 9615)	
Residence (%)	Urban	4059 (33.9)	774 (19.1)	3285 (80.9)	0.311
	Rural	7899 (66.1)	1569 (19.9)	6330 (80.1)	
Gender (%)	Male	5046 (42.2)	1079 (21.4)	3967 (78.6)	<0.001
	Female	6912 (57.8)	1264 (18.3)	5648 (81.7)	
Age (%)	65~85	4224 (35.3)	2020 (47.8)	2204 (52.2)	<0.001
	85~100	5170 (43.2)	287 (5.6)	4883 (94.4)	
	≥100	2564 (21.4)	36 (1.4)	2528 (98.6)	
Education level (%)	Uneducated	7850 (65.6)	1145 (14.6)	6705 (85.4)	<0.001
	Educated	4080 (34.1)	882 (28.1)	2261 (71.9)	
	Missing	28 (0.3)	3 (10.7)	25 (89.3)	
Marital status (%)	Currently married	3386 (28.3)	1354 (40.0)	2032 (60.0)	<0.001
	Others	8572 (71.7)	989 (11.5)	7583 (88.5)	
Smoking status (%)	Never smoked	7968 (66.6)	1515 (19.0)	6453 (81.0)	<0.001
	Former smoker	1910 (16.0)	316 (16.5)	1594 (83.5)	
	Current smoker	2080 (17.4)	512 (24.6)	1568 (75.4)	
Alcohol consumption (%)	Never drank	8181 (68.4)	1550 (18.9)	6631 (81.1)	<0.001
	Former drinker	1678 (14.0)	291 (17.3)	1387 (82.7)	
	Current drinker	2099 (17.6)	502 (23.9)	1597 (76.1)	
Exercise status (%)	Never	7475 (62.5)	1320 (17.7)	6155 (82.3)	<0.001
	Former	1527 (12.8)	196 (12.8)	1331 (87.2)	
	Current	2956 (24.7)	827 (28.0)	2129 (72.0)	
Sleep duration (%)	≤6 h	3033 (25.3)	704 (23.2)	2329 (76.8)	<0.001
	6~8 h	4144 (34.7)	1028 (24.8)	3116 (75.2)	
	>8 h	4721 (39.5)	609 (12.9)	4112 (87.1)	
	Missing	60 (0.5)	2 (3.3)	58 (96.7)	
MMSE (%)	Severe cognitive impairment	129 (1.1)	2 (1.6)	127 (98.4)	<0.001
	Moderate cognitive impairment	1058 (8.8)	87 (8.2)	971 (91.8)	
	Mild cognitive impairment	2066 (17.3)	445 (21.5)	1621 (78.5)	
	Normal	4504 (37.7)	1611 (35.8)	2893 (64.2)	
	Missing	4201 (35.1)	198 (4.7)	4003 (95.3)	
BMI (%)	<18.5	4038 (33.8)	513 (12.7)	3525 (87.3)	<0.001
	18.5–23.9	6103 (51.0)	1321 (21.6)	4782 (78.4)	
	24–27.9	1169 (9.8)	393 (33.6)	776 (66.4)	
	≥28	293 (2.5)	103 (35.2)	190 (64.8)	
	Missing	355 (3.0)	13 (3.7)	342 (96.3)	
Disease (%)	No	9211 (77.0)	1735 (18.8)	7476 (81.2)	<0.001
	Yes	2747 (23.0)	608 (22.1)	2139 (77.9)	

* *p*-values between living and dead participants were obtained using the chi-square test.

**Table 2:** Factor loads for each food group in four dietary patterns *.

	Milk–Egg–Sugar Pattern	Carnivorous Pattern	Healthy Pattern	Northeastern Pattern
Dairy	0.699			
Eggs	0.629			
Sugar	0.615			
Soybeans	0.440			
Mushrooms or Algae	0.435			
Meat		0.784		
Seafood		0.776		
Amount of Staple Foods			0.578	
Fresh Fruits			0.536	
Nuts			0.526	
Fresh Vegetables			0.505	
Mushrooms or Algae			0.448	
Salty Vegetables				0.772
Garlic				0.618
Tea				0.503
Explained variance (%)	13.566	11.585	10.579	10.522

* Only the types of foods that characterize each dietary pattern (FL ≥ 0.4) are presented in the table.

**Table 3:** Hazard ratios of all-cause mortality according to quartiles of four dietary pattern scores (95% CI) based on gender.

	Q1	Q2	*p*	Q3	*p*	Q4 *	*p*	*p*-Trend	Global
Males									
(N = 5046)									
Milk–Egg–Sugar Pattern									
Alive (N (%))	264 (20.3)	282 (21.9)		265 (24.6)		268 (24.8)			1079 (21.4)
Dead (N (%))	1038 (79.7)	1005 (25.3)		932 (23.5)		992 (25.0)			3967 (78.6)
Model 1 ^a^ (HR (95% CI))	reference	0.90 (0.83–0.98)	0.020	0.89 (0.82–0.97)	0.011	0.90 (0.83–0.98)	0.019	0.023	0.97 (0.94–0.99)
Model 2 ^b^ (HR (95% CI))	reference	0.91 (0.83–0.99)	0.029	0.91 (0.83–0.99)	0.038	0.94 (0.86–1.03)	0.157	0.181	0.98 (0.95–1.01)
Model 3 ^c^ (HR (95% CI))	reference	0.91 (0.84–0.99)	0.045	0.93 (0.85–1.01)	0.091	0.96 (0.88–1.06)	0.448	0.414	0.99 (0.96–1.02)
Carnivorous Pattern									
Alive (N (%))	234 (19.2)	251 (20.0)		274 (21.7)		320 (24.5)			1079 (21.4)
Dead (N (%))	987 (80.8)	1007 (80.0)		988 (78.3)		985 (75.5)			3967 (78.6)
Model 1 ^a^ (HR (95% CI))	reference	0.96 (0.88–1.05)	0.333	0.91 (0.83–0.99)	0.028	0.80 (0.73–0.87)	<0.001	<0.001	0.93 (0.90–0.96)
Model 2 ^b^ (HR (95% CI))	reference	0.99 (0.9–1.08)	0.739	0.91 (0.84–0.99)	0.050	0.83 (0.76–0.91)	<0.001	<0.001	0.94 (0.91–0.97)
Model 3 ^c^ (HR (95% CI))	reference	0.98 (0.9–1.07)	0.697	0.92 (0.84–1.01)	0.086	0.84 (0.77–0.93)	<0.001	<0.001	0.94 (0.92–0.97)
Healthy Pattern									
Alive (N (%))	99 (10.3)	189 (16.0)		310 (23.2)		481 (30.7)			1079 (21.4)
Dead (N (%))	863 (89.7)	993 (84.0)		1027 (76.8)		1084 (69.3)			3967 (78.6)
Model 1 ^a^ (HR (95% CI))	reference	0.84 (0.77–0.92)	<0.001	0.73 (0.67–0.80)	<0.001	0.63 (0.57–0.68)	<0.001	<0.001	0.86 (0.83–0.88)
Model 2 ^b^ (HR (95% CI))	reference	0.85 (0.78–0.94)	<0.001	0.75 (0.68–0.82)	<0.001	0.65 (0.59–0.71)	<0.001	<0.001	0.87 (0.84–0.89)
Model 3 ^c^ (HR (95% CI))	reference	0.85 (0.77–0.93)	<0.001	0.74 (0.68–0.82)	<0.001	0.65 (0.59–0.72)	<0.001	<0.001	0.87 (0.84–0.89)
Northeastern Pattern									
Alive (N (%))	132 (13.1)	241 (19.8)		297 (22.2)		409 (27.6)			1079 (21.4)
Dead (N (%))	874 (86.9)	977 (80.2)		1042 (77.8)		1074 (72.4)			3967 (78.6)
Model 1 ^a^ (HR (95% CI))	reference	0.82 (0.75–0.90)	<0.001	0.80 (0.73–0.87)	<0.001	0.77 (0.71–0.85)	<0.001	<0.001	0.93 (0.90–0.95)
Model 2 ^b^ (HR (95% CI))	reference	0.83 (0.75–0.90)	<0.001	0.81 (0.74–0.89)	<0.001	0.80 (0.73–0.88)	<0.001	<0.001	0.94 (0.91–0.96)
Model 3 ^c^ (HR (95% CI))	reference	0.84 (0.76–0.92)	0.001	0.81 (0.74–0.89)	<0.001	0.82 (0.75–0.90)	0.006	<0.001	0.94 (0.92–0.97)
Females									
(N = 6912)									
Milk–Egg–Sugar Pattern									
Alive (N (%))	343 (20.3)	313 (18.4)		310 (17.3)		298 (17.2)			1264 (18.3)
Dead (N (%))	1345 (79.7)	1387 (81.6)		1484 (82.7)		1432 (82.8)			5648 (81.7)
Model 1 ^a^ (HR (95% CI))	reference	1.04 (0.96–1.12)	0.350	1.04 (0.97–1.12)	0.288	1.05 (0.98–1.14)	0.165	0.072	1.02 (0.99–1.05)
Model 2 ^b^ (HR (95% CI))	reference	1.05 (0.98–1.14)	0.177	1.06 (0.98–1.14)	0.150	1.08 (1.00–1.16)	0.055	0.065	1.02 (0.99–1.05)
Model 3 ^c^ (HR (95% CI))	reference	1.05 (0.98–1.14)	0.186	1.06 (0.99–1.15)	0.112	1.09 (1.01–1.18)	0.026	0.029	1.03 (1.01–1.05)
Carnivorous Pattern									
Alive (N (%))	305 (17.2)	307 (17.7)		324 (18.8)		328 (19.5)			1264 (18.3)
Dead (N (%))	1464 (82.8)	1424 (82.3)		1403 (81.2)		1357 (80.5)			5648 (81.7)
Model 1 ^a^ (HR (95% CI))	reference	0.97 (0.9–1.05)	0.440	0.95 (0.88–1.02)	0.173	0.96 (0.89–1.03)	0.232	0.183	0.98 (0.96–1.01)
Model 2 ^b^ (HR (95% CI))	reference	0.99 (0.92–1.06)	0.743	0.98 (0.91–1.05)	0.543	0.98 (0.91–1.05)	0.535	0.495	0.99 (0.97–1.02)
Model 3 ^c^ (HR (95% CI))	reference	0.99 (0.92–1.06)	0.743	0.98 (0.91–1.06)	0.600	0.99 (0.91–1.06)	0.727	0.688	1.00 (0.97–1.02)
Healthy Pattern									
Alive (N (%))	229 (11.3)	294 (16.3)		328 (19.9)		413 (29.0)			1264 (18.3)
Dead (N (%))	1798 (88.7)	1514 (83.7)		1324 (80.1)		1012 (71.0)			5648 (81.7)
Model 1 ^a^ (HR (95% CI))	reference	0.92 (0.86–0.99)	0.019	0.91 (0.85–0.98)	0.011	0.79 (0.73–0.86)	<0.001	<0.001	0.93 (0.91–0.96)
Model 2 ^b^ (HR (95% CI))	reference	0.93 (0.86–0.99)	0.029	0.92 (0.86–0.99)	0.033	0.83 (0.77–0.90)	<0.001	<0.001	0.95 (0.92–0.97)
Model 3 ^c^ (HR (95% CI))	reference	0.92 (0.85–0.98)	0.013	0.92 (0.85–0.98)	0.017	0.83 (0.76–0.90)	<0.001	<0.001	0.95 (0.92–0.97)
Northeastern Pattern									
Alive (N (%))	247 (12.4)	271 (15.3)		360 (21.8)		386 (25.6)			1264 (18.3)
Dead (N (%))	1737 (87.6)	1500 (84.7)		1290 (78.2)		1121 (74.4)			5648 (81.7)
Model 1 ^a^ (HR (95% CI))	reference	0.97 (0.90–1.04)	0.358	0.88 (0.82–0.95)	0.001	0.87 (0.80–0.93)	<0.001	<0.001	0.95 (0.93–0.97)
Model 2 ^b^ (HR (95% CI))	reference	0.97 (0.90–1.04)	0.327	0.88 (0.82–0.95)	0.001	0.88 (0.82–0.95)	0.001	<0.001	0.95 (0.93–0.98)
Model 3 ^c^ (HR (95% CI))	reference	0.97 (0.9–1.04)	0.385	0.89 (0.83–0.96)	0.002	0.89 (0.82–0.96)	0.003	<0.001	0.96 (0.93–0.98)

* Q4 is the most adherent to the dietary pattern. ^a^: Model 1 = dietary pattern+ age. ^b^: Model 2 = dietary pattern + age + residence + education level + marital status. ^c^: Model 3 = dietary pattern + age + residence + education level + marital status + smoking + alcohol assumption + exercise status +sleep duration + cognitive function + BMI + disease.

## Data Availability

The data presented in this study are available on request from the corresponding author due to privacy.

## Supplementary Materials

The following supporting information can be downloaded at: https://www.mdpi.com/article/10.3390/nu16111605/s1, Table S1: Baseline characteristics of the CLHLS according to quintiles of four dietary pattern scores, Table S2: Characterization of dietary intake frequency among older adults in the CLHLS, grouped by final status, Table S3: Hazard ratios of all-cause mortality according to quartiles of four dietary pattern scores (95% CI) based on age group. Figure S1: Scree plot.
