# Supplementary material for: Association between Dietary Patterns and All-Cause Mortality in the Chinese Old: Analysis of the Chinese Longitudinal Healthy Longevity Survey Cohort
Source: Nutrients. 2024 May 24;16(11):1605. doi: 10.3390/nu16111605 (PMC11174105; doi:10.3390/nu16111605)
Supplement: Supplementary file 1 [file nutrients-16-01605-s001.zip › nutrients-2992694-supplementary.pdf]

Table S1. Baseline characteristics of the CLHLS according to quartiles of four dietary pattern scores

	Level	Overall (N=11,958)	Q1 (N=2,990)	Q2 (N=2,987)	Q3 (N=2,991)	Q4* (N=2,990)	P**
**Milk-egg-sugar pattern**							
Residence (%)	Urban	4059 (33.9)	716 (23.9)	915 (30.6)	976 (32.6)	1452 (48.6)	<0.001
	Rural	7899 (66.1)	2274 (76.1)	2072 (69.4)	2015 (67.4)	1538 (51.4)	
Sex (%)	Men	4224 (35.3)	1149 (38.4)	1105 (37.0)	1020 (34.1)	950 (31.8)	<0.001
	Women	5170 (43.2)	1322 (44.2)	1346 (45.1)	1261 (42.2)	1241 (41.5)	
Age (%)	65~85	2564 (21.4)	519 (17.4)	536 (17.9)	710 (23.7)	799 (26.7)	
	85~100	5046 (42.2)	1302 (43.5)	1287 (43.1)	1197 (40.0)	1260 (42.1)	0.029
	≥100	6912 (57.8)	1688 (56.5)	1700 (56.9)	1794 (60.0)	1730 (57.9)	
Educational level (%)	Uneducated	7850 (65.8)	2061 (69.1)	1964 (65.8)	1975 (66.2)	1850 (62.0)	<0.001
	Educated	4080 (34.2)	921 (30.9)	1019 (34.2)	1007 (33.8)	1133 (37.9)	
Marital status (%)	Currently married	3386 (28.3)	848 (28.4)	875 (29.3)	810 (27.1)	853 (28.5)	0.293
	Others	8572 (71.7)	2142 (71.6)	2112 (70.7)	2181 (72.9)	2137 (71.5)	
Smoke (%)	Never smoked	7968 (66.6)	2018 (67.5)	1999 (66.9)	2005 (67.0)	1946 (65.1)	0.007
	Former smoker	1910 (16.0)	434 (14.5)	462 (15.5)	468 (15.6)	546 (18.3)	
	Current smoker	2080 (17.4)	538 (18.0)	526 (17.6)	518 (17.3)	498 (16.7)	
Alcohol consumption (%)	Never drink	8181 (68.4)	2123 (71.0)	2043 (68.4)	2036 (68.1)	1979 (66.2)	0.001
	Former drinker	1678 (14.0)	417 (13.9)	421 (14.1)	409 (13.7)	431 (14.4)	
	Current smoker	2099 (17.6)	450 (15.1)	523 (17.5)	546 (18.3)	580 (19.4)	
Exercise status (%)	Never	7475 (62.5)	2106 (70.4)	1949 (65.2)	1876 (62.7)	1544 (51.6)	<0.001
	Former	1527 (12.8)	345 (11.5)	363 (12.2)	382 (12.8)	437 (14.6)	
	Current	2956 (24.7)	539 (18.0)	675 (22.6)	733 (24.5)	1009 (33.7)	

Sleep (%)	≤6h	3033 (25.5)	948 (31.8)	734 (24.7)	741 (24.9)	610 (20.5)	<0.001
	6~8h	4144 (34.8)	1118 (37.6)	1083 (36.4)	954 (32.1)	989 (33.3)	
	>8h	4721 (39.7)	911 (30.6)	1158 (38.9)	1278 (43.0)	1374 (46.2)	
MMSE (%)	Severe cognitive impairment	129 (1.1)	44 (1.5)	31 (1.0)	28 (0.9)	26 (0.9)	0.073
	Moderate cognitive impairment	1058 (8.8)	244 (8.2)	287 (9.6)	296 (9.9)	231 (7.7)	
	Mild cognitive impairment	2066 (17.3)	531 (17.8)	513 (17.2)	511 (17.1)	511 (17.1)	
	Normal	4504 (37.7)	1129 (37.8)	1107 (37.1)	1116 (37.3)	1152 (38.5)	
	Missing	4201 (35.1)	1042 (34.8)	1049 (35.1)	1040 (34.8)	1070 (35.8)	
BMI (%)	＜18.5	4038 (33.8)	1253 (41.9)	1031 (34.5)	954 (31.9)	800 (26.8)	<0.001
	18.5-23.9	6103 (51.0)	1460 (48.8)	1503 (50.3)	1579 (52.8)	1561 (52.2)	
	24-27.9	1169 (9.8)	178 (6.0)	319 (10.7)	287 (9.6)	385 (12.9)	
	⩾28	293 (2.5)	47 (1.6)	60 (2.0)	75 (2.5)	111 (3.7)	
	Missing	355 (3.0)	52 (1.7)	74 (2.5)	96 (3.2)	133 (4.4)	
Disease (%)	No	9211 (77.0)	2335 (78.1)	2312 (77.4)	2343 (78.3)	2221 (74.3)	<0.001
	Yes	2747 (23.0)	655 (21.9)	675 (22.6)	648 (21.7)	769 (25.7)	
**Carnivorous pattern**							
Residence (%)	Urban	4059 (33.9)	691 (23.1)	863 (28.9)	1068 (35.7)	1437 (48.1)	<0.001
	Rural	7899 (66.1)	2299 (76.9)	2126 (71.1)	1921 (64.3)	1553 (51.9)	
Sex (%)	Men	4224 (35.3)	1057 (35.4)	1052 (35.2)	1084 (36.3)	1031 (34.5)	0.034
	Women	5170 (43.2)	1343 (44.9)	1304 (43.6)	1259 (42.1)	1264 (42.3)	
Age (%)	65~85	2564 (21.4)	590 (19.7)	633 (21.2)	646 (21.6)	695 (23.2)	
	85~100	5046 (42.2)	1221 (40.8)	1258 (42.1)	1262 (42.2)	1305 (43.6)	0.183
	≥100	6912 (57.8)	1769 (59.2)	1731 (57.9)	1727 (57.8)	1685 (56.4)	

Educational level (%)	Uneducated	7850 (65.8)	2179 (73.0)	2050 (68.8)	1920 (64.4)	1701 (57.0)	<0.001
	Educated	4080 (34.2)	807 (27.0)	929 (31.2)	1062 (35.6)	1282 (42.9)	
Marital status (%)	Currently married	3386 (28.3)	739 (24.7)	900 (30.1)	856 (28.6)	891 (29.8)	<0.001
	Others	8572 (71.7)	2251 (75.3)	2089 (69.9)	2133 (71.4)	2099 (70.2)	
Smoke (%)	Never smoked	7968 (66.6)	2020 (67.6)	1979 (66.2)	2012 (67.3)	1957 (65.5)	0.107
	Former smoker	1910 (16.0)	484 (16.2)	455 (15.2)	458 (15.3)	513 (17.2)	
	Current smoker	2080 (17.4)	486 (16.3)	555 (18.6)	519 (17.4)	520 (17.4)	
Alcohol consumption (%)	Never drink	8181 (68.4)	2163 (72.3)	2030 (67.9)	1997 (66.8)	1991 (66.6)	<0.001
	Former drinker	1678 (14.0)	424 (14.2)	429 (14.4)	418 (14.0)	407 (13.6)	
	Current smoker	2099 (17.6)	403 (13.5)	530 (17.7)	574 (19.2)	592 (19.8)	
Exercise status (%)	Never	7475 (62.5)	2102 (70.3)	1954 (65.4)	1827 (61.1)	1592 (53.2)	<0.001
	Former	1527 (12.8)	354 (11.8)	354 (11.8)	397 (13.3)	422 (14.1)	
	Current	2956 (24.7)	534 (17.9)	681 (22.8)	765 (25.6)	976 (32.6)	
Sleep (%)	≤6h	3033 (25.5)	895 (30.2)	709 (23.8)	720 (24.2)	709 (23.8)	<0.001
	6~8h	4144 (34.8)	1050 (35.4)	1030 (34.6)	1006 (33.8)	1058 (35.5)	
	>8h	4721 (39.7)	1022 (34.4)	1235 (41.5)	1252 (42.0)	1212 (40.7)	
MMSE (%)	Severe cognitive impairment	129 (1.1)	36 (1.2)	43 (1.4)	27 (0.9)	23 (0.8)	<0.001
	Moderate cognitive impairment	1058 (8.8)	343 (11.5)	278 (9.3)	254 (8.5)	183 (6.1)	
	Mild cognitive impairment	2066 (17.3)	566 (18.9)	526 (17.6)	538 (18.0)	436 (14.6)	
	Normal	4504 (37.7)	862 (28.8)	1023 (34.2)	1209 (40.4)	1410 (47.2)	
	Missing	4201 (35.1)	1183 (39.6)	1119 (37.4)	961 (32.2)	938 (31.4)	
BMI (%)	＜18.5	4038 (33.8)	1051 (35.2)	1031 (34.5)	1031 (34.5)	925 (30.9)	0.007
	18.5-23.9	6103 (51.0)	1521 (50.9)	1519 (50.8)	1511 (50.6)	1552 (51.9)	

	24-27.9	1169 (9.8)	270 (9.0)	290 (9.7)	273 (9.1)	336 (11.2)	
	⩾28	293 (2.5)	67 (2.2)	68 (2.3)	86 (2.9)	72 (2.4)	
	Missing	355 (3.0)	81 (2.7)	81 (2.7)	88 (2.9)	105 (3.5)	
Disease (%)	No	9211 (77.0)	2306 (77.1)	2317 (77.5)	2325 (77.8)	2263 (75.7)	0.218
	Yes	2747 (23.0)	684 (22.9)	672 (22.5)	664 (22.2)	727 (24.3)	
**Healthy pattern**							
Residence (%)	Urban	4059 (33.9)	806 (27.0)	883 (29.5)	988 (33.1)	1382 (46.2)	<0.001
	Rural	7899 (66.1)	2183 (73.0)	2107 (70.5)	2001 (66.9)	1608 (53.8)	
Sex (%)	Men	4224 (35.3)	671 (22.4)	962 (32.2)	1160 (38.8)	1431 (47.9)	<0.001
	Women	5170 (43.2)	1439 (48.1)	1387 (46.4)	1261 (42.2)	1083 (36.2)	
Age (%)	65~85	2564 (21.4)	879 (29.4)	641 (21.4)	568 (19.0)	476 (15.9)	
	85~100	5046 (42.2)	962 (32.2)	1182 (39.5)	1337 (44.7)	1565 (52.3)	<0.001
	≥100	6912 (57.8)	2027 (67.8)	1808 (60.5)	1652 (55.3)	1425 (47.7)	
Educational level (%)	Uneducated	7850 (65.8)	2336 (78.4)	2103 (70.5)	1860 (62.4)	1551 (52.0)	<0.001
	Educated	4080 (34.2)	644 (21.6)	881 (29.5)	1121 (37.6)	1434 (48.0)	
Marital status (%)	Currently married	3386 (28.3)	540 (18.1)	762 (25.5)	874 (29.2)	1210 (40.5)	<0.001
	Others	8572 (71.7)	2449 (81.9)	2228 (74.5)	2115 (70.8)	1780 (59.5)	
Smoke (%)	Never smoked	7968 (66.6)	2216 (74.1)	2046 (68.4)	1935 (64.7)	1771 (59.2)	<0.001
	Former smoker	1910 (16.0)	406 (13.6)	433 (14.5)	483 (16.2)	588 (19.7)	
	Current smoker	2080 (17.4)	367 (12.3)	511 (17.1)	571 (19.1)	631 (21.1)	
Alcohol consumption (%)	Never drink	8181 (68.4)	2218 (74.2)	2088 (69.8)	2015 (67.4)	1860 (62.2)	<0.001
	Former drinker	1678 (14.0)	378 (12.6)	412 (13.8)	418 (14.0)	470 (15.7)	
	Current smoker	2099 (17.6)	393 (13.1)	490 (16.4)	556 (18.6)	660 (22.1)	

Exercise status (%)	Never	7475 (62.5)	2092 (70.0)	1993 (66.7)	1852 (62.0)	1538 (51.4)	<0.001
	Former	1527 (12.8)	413 (13.8)	373 (12.5)	366 (12.2)	375 (12.5)	
	Current	2956 (24.7)	484 (16.2)	624 (20.9)	771 (25.8)	1077 (36.0)	
Sleep (%)	≤6h	3033 (25.5)	972 (32.8)	734 (24.7)	689 (23.1)	638 (21.4)	<0.001
	6~8h	4144 (34.8)	847 (28.6)	1070 (36.0)	1077 (36.2)	1150 (38.6)	
	>8h	4721 (39.7)	1145 (38.6)	1172 (39.4)	1211 (40.7)	1193 (40.0)	
MMSE (%)	Severe cognitive impairment	129 (1.1)	69 (2.3)	24 (0.8)	18 (0.6)	18 (0.6)	<0.001
	Moderate cognitive impairment	1058 (8.8)	375 (12.5)	288 (9.6)	219 (7.3)	176 (5.9)	
	Mild cognitive impairment	2066 (17.3)	483 (16.2)	565 (18.9)	528 (17.7)	490 (16.4)	
	Normal	4504 (37.7)	613 (20.5)	977 (32.7)	1292 (43.2)	1622 (54.2)	
	Missing	4201 (35.1)	1449 (48.5)	1136 (38.0)	932 (31.2)	684 (22.9)	
BMI (%)	＜18.5	4038 (33.8)	1371 (45.9)	1109 (37.1)	914 (30.6)	644 (21.5)	<0.001
	18.5-23.9	6103 (51.0)	1304 (43.6)	1520 (50.8)	1633 (54.6)	1646 (55.1)	
	24-27.9	1169 (9.8)	167 (5.6)	236 (7.9)	280 (9.4)	486 (16.3)	
	⩾28	293 (2.5)	38 (1.3)	51 (1.7)	77 (2.6)	127 (4.2)	
	Missing	355 (3.0)	109 (3.6)	74 (2.5)	85 (2.8)	87 (2.9)	
Disease (%)	No	9211 (77.0)	2367 (79.2)	2350 (78.6)	2306 (77.1)	2188 (73.2)	<0.001
	Yes	2747 (23.0)	622 (20.8)	640 (21.4)	683 (22.9)	802 (26.8)	
**Northeastern pattern**							
Residence (%)	Urban	4059 (33.9)	841 (28.1)	1040 (34.8)	1098 (36.7)	1080 (36.1)	<0.001
	Rural	7899 (66.1)	2149 (71.9)	1949 (65.2)	1891 (63.3)	1910 (63.9)	
Sex (%)	Men	4224 (35.3)	734 (24.5)	946 (31.6)	1124 (37.6)	1420 (47.5)	<0.001
	Women	5170 (43.2)	1353 (45.3)	1351 (45.2)	1313 (43.9)	1153 (38.6)	

Age (%)	65~85	2564 (21.4)	903 (30.2)	692 (23.2)	552 (18.5)	417 (13.9)	
	85~100	5046 (42.2)	1006 (33.6)	1218 (40.7)	1339 (44.8)	1483 (49.6)	<0.001
	≥100	6912 (57.8)	1984 (66.4)	1771 (59.3)	1650 (55.2)	1507 (50.4)	
Educational level (%)	Uneducated	7850 (65.8)	2172 (72.8)	2010 (67.4)	1946 (65.3)	1722 (57.7)	<0.001
	Educated	4080 (34.2)	811 (27.2)	971 (32.6)	1035 (34.7)	1263 (42.3)	
Marital status (%)	Currently married	3386 (28.3)	589 (19.7)	763 (25.5)	904 (30.2)	1130 (37.8)	<0.001
	Others	8572 (71.7)	2401 (80.3)	2226 (74.5)	2085 (69.8)	1860 (62.2)	
Smoke (%)	Never smoked	7968 (66.6)	2220 (74.2)	2063 (69.0)	1906 (63.8)	1779 (59.5)	<0.001
	Former smoker	1910 (16.0)	393 (13.1)	483 (16.2)	490 (16.4)	544 (18.2)	
	Current smoker	2080 (17.4)	377 (12.6)	443 (14.8)	593 (19.8)	667 (22.3)	
Alcohol consumption (%)	Never drink	8181 (68.4)	2247 (75.2)	2132 (71.3)	1974 (66.0)	1828 (61.1)	<0.001
	Former drinker	1678 (14.0)	365 (12.2)	386 (12.9)	453 (15.2)	474 (15.9)	
	Current smoker	2099 (17.6)	378 (12.6)	471 (15.8)	562 (18.8)	688 (23.0)	
Exercise status (%)	Never	7475 (62.5)	2079 (69.5)	1884 (63.0)	1793 (60.0)	1719 (57.5)	<0.001
	Former	1527 (12.8)	376 (12.6)	428 (14.3)	371 (12.4)	352 (11.8)	
	Current	2956 (24.7)	535 (17.9)	677 (22.6)	825 (27.6)	919 (30.7)	
Sleep (%)	≤6h	3033 (25.5)	856 (28.9)	749 (25.2)	711 (23.9)	717 (24.0)	<0.001
	6~8h	4144 (34.8)	1013 (34.2)	994 (33.4)	1006 (33.8)	1131 (37.9)	
	>8h	4721 (39.7)	1095 (36.9)	1230 (41.4)	1258 (42.3)	1138 (38.1)	
MMSE (%)	Severe cognitive impairment	129 (1.1)	53 (1.8)	27 (0.9)	27 (0.9)	22 (0.7)	<0.001
	Moderate cognitive impairment	1058 (8.8)	272 (9.1)	260 (8.7)	261 (8.7)	265 (8.9)	
	Mild cognitive impairment	2066 (17.3)	440 (14.7)	487 (16.3)	562 (18.8)	577 (19.3)	
	Normal	4504 (37.7)	856 (28.6)	1011 (33.8)	1166 (39.0)	1471 (49.2)	

	Missing	4201 (35.1)	1369 (45.8)	1204 (40.3)	973 (32.6)	655 (21.9)	
BMI (%)	＜18.5	4038 (33.8)	1248 (41.7)	1068 (35.7)	950 (31.8)	772 (25.8)	<0.001
	18.5-23.9	6103 (51.0)	1358 (45.4)	1507 (50.4)	1538 (51.5)	1700 (56.9)	
	24-27.9	1169 (9.8)	209 (7.0)	257 (8.6)	332 (11.1)	371 (12.4)	
	⩾28	293 (2.5)	48 (1.6)	59 (2.0)	83 (2.8)	103 (3.4)	
	Missing	355 (3.0)	127 (4.2)	98 (3.3)	86 (2.9)	44 (1.5)	
Disease (%)	No	9211 (77.0)	2327 (77.8)	2326 (77.8)	2274 (76.1)	2284 (76.4)	0.226
	Yes	2747 (23.0)	663 (22.2)	663 (22.2)	715 (23.9)	706 (23.6)	

* Q4 is the most adherent to that dietary pattern.

**p values between participants in different quartiles of four dietary pattern scores were obtained by chi square test.

Table S2. Characterization of dietary intake frequency among older adults in the CLHLS grouped by final status

	Level	Total	Status		P*
			Alive	Dead	
N		11958	2343	9615	
Amount of Staple (%)	0-4 taels/day	2110 (17.6)	228 (10.8)	1882 (89.2)	<0.001
	4-6 taels/day	3799 (31.8)	576 (15.2)	3223 (84.8)	
	6-8 taels/day	3048 (25.5)	582 (19.1)	2466 (80.1)	
	≥8 taels/day	3001 (25.1)	957 (31.9)	2044 (68.1)	
Fresh Fruit (%)	Seldom or Not	3197 (26.7)	460 (14.4)	2737 (85.6)	<0.001
	Sometime	4486 (37.5)	896 (20.0)	3590 (80.0)	
	Usually	2983 (24.9)	711 (23.8)	2272 (76.2)	
	(Nearly) Everyday	1292 (10.8)	276 (7.1)	1016 (92.9)	
Fresh Vegetable (%)	Seldom or Not	367 (3.1)	26 (1.1)	341 (3.5)	<0.001
	Sometime	1182 (9.9)	171 (14.5)	1011 (85.5)	
	Usually	3131 (26.2)	586 (18.7)	2545 (81.3)	
	(Nearly) Everyday	7278 (60.9)	1560 (21.4)	5718 (78.6)	
Meat (%)	Seldom or Not	2600 (21.7)	464 (17.8)	2136 (82.2)	0.003
	Sometime	1386 (11.6)	309 (22.3)	1077 (77.7)	
	Usually	4673 (39.1)	948 (20.3)	3725 (79.7)	
	(Nearly) Everyday	3299 (27.6)	622 (18.9)	2677 (81.1)	
Seafood (%)	Seldom or Not	5244 (43.9)	880 (16.8)	4364 (83.2)	<0.001
	Sometime	2361 (19.7)	499 (21.1)	1862 (78.9)	

	Usually	3560 (29.8)	793 (22.3)	2767 (77.7)	
	(Nearly) Everyday	793 (6.6)	171 (21.6)	622 (78.4)	
Eggs (%)	Seldom or Not	2286 (19.1)	457 (20.0)	1829 (80.0)	<0.001
	Sometime	1433 (12.0)	289 (20.2)	1144 (79.8)	
	Usually	4163 (34.8)	890 (21.4)	3273 (78.6)	
	(Nearly) Everyday	4076 (34.1)	707 (17.3)	3369 (82.7)	
Soybeans (%)	Seldom or Not	3661 (30.6)	685 (18.7)	2976 (81.3)	0.416
	Sometime	1864 (15.6)	370 (19.8)	1494 (80.2)	
	Usually	4527 (37.9)	913 (20.2)	3614 (79.8)	
	(Nearly) Everyday	1906 (15.9)	375 (19.7)	1531 (80.3)	
Salty Vegetables (%)	Seldom or Not	6872 (57.5)	1118 (16.3)	5754 (83.7)	<0.001
	Sometime	1029 (8.6)	243 (23.6)	786 (76.4)	
	Usually	1860 (15.6)	435 (23.4)	1425 (76.6)	
	(Nearly) Everyday	2197 (18.4)	547 (24.9)	1650 (75.1)	
Sugar (%)	Seldom or Not	6222 (52.0)	1350 (21.7)	4872 (78.3)	<0.001
	Sometime	1257 (10.5)	266 (21.2)	991 (78.8)	
	Usually	2341 (19.6)	459 (19.6)	1882 (80.4)	
	(Nearly) Everyday	2138 (17.9)	268 (12.5)	1870 (87.5)	
Tea (%)	Seldom or Not	7576 (63.4)	1315 (17.4)	6261 (82.6)	<0.001
	Sometime	304 (2.5)	61 (20.1)	243 (79.9)	
	Usually	620 (5.2)	132 (21.3)	488 (78.7)	
	(Nearly) Everyday	3458 (28.9)	835 (24.1)	2623 (75.9)	
Garlic (%)	Seldom or Not	6046 (50.6)	998 (16.5)	5048 (83.5)	<0.001

	Sometime	1203 (10.1)	250 (20.8)	953 (79.2)	
	Usually	2470 (20.7)	544 (22.0)	1926 (78.0)	
	(Nearly) Everyday	2239 (18.7)	551 (24.6)	1688 (75.4)	
Dairy (%)	Seldom or Not	8006 (67.0)	1643 (20.5)	6363 (79.5)	<0.001
	Sometime	720 (6.0)	144 (20.0)	576 (80.0)	
	Usually	1196 (10.0)	230 (19.2)	966 (80.8)	
	(Nearly) Everyday	2036 (17.0)	326 (16.0)	1710 (84.0)	
Nuts (%)	Seldom or Not	10043 (84.0)	1720 (17.1)	8323 (82.9)	<0.001
	Sometime	818 (6.8)	261 (31.9)	557 (68.1)	
	Usually	779 (6.5)	260 (33.4)	519 (66.6)	
	(Nearly) Everyday	318 (2.7)	102 (32.1)	216 (67.9)	
Mushrooms or Algae (%)	Seldom or Not	9360 (78.3)	1739 (18.6)	7621 (81.4)	<0.001
	Sometime	1412 (11.8)	296 (21.0)	1116 (79.0)	
	Usually	1022 (8.5)	263 (25.7)	759 (74.3)	
	(Nearly) Everyday	164 (1.4)	45 (27.4)	119 (72.6)	

*p values between alive and dead participants were obtained by chi square test.

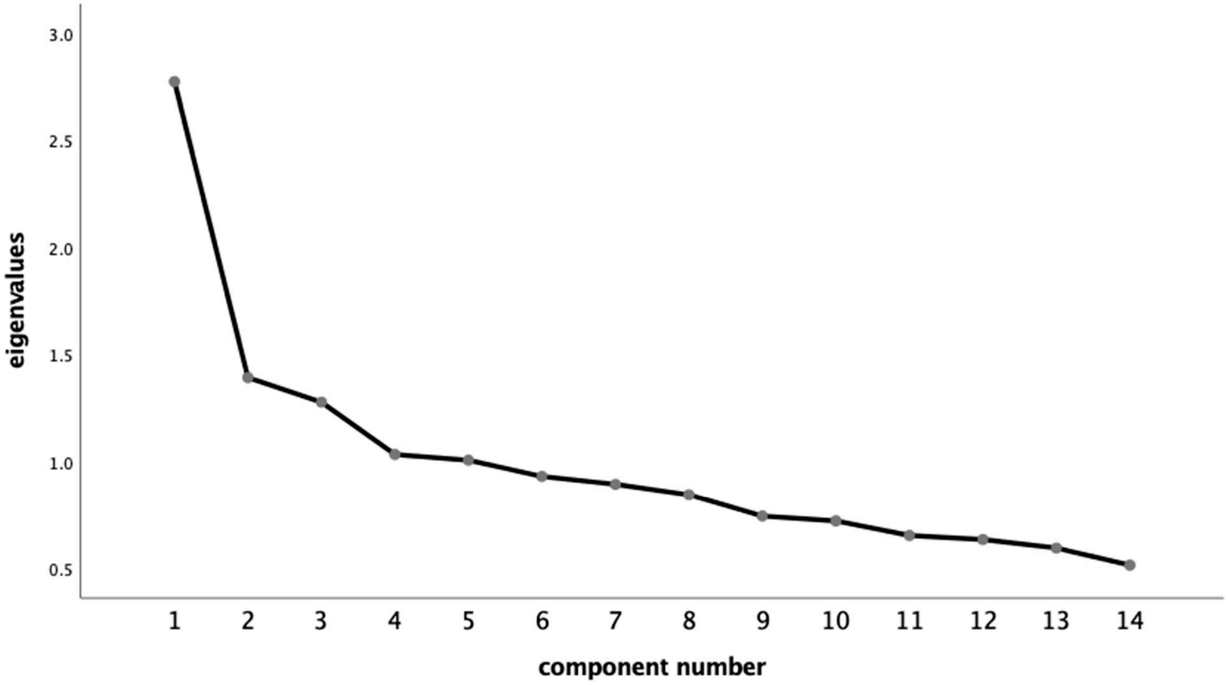

Figure S1. Scree plot

Table S3. Hazard ratios of all-cause mortality according to quartiles of four dietary pattern scores (95% CI) by age group*.

	Q1	Q2	P	Q3	P	Q4	P	P-trend	GLOBAL
**65~85 (N=4224)**									
**MILK-EGG-SUGAR PATTERN**	reference								
**CARNIVOROUS PATTERN**	reference					0.84(0.74-0.96)	0.007	0.005	0.96(0.91-0.98)
**HEALTHY PATTERN**	reference	0.83(0.73-0.95)	0.005	0.69(0.61-0.78)	<0.001	0.58(0.50-0.66)	<0.001	<0.001	0.83(0.80-0.87)
**NORTHEASTERN PATTERN**	reference			0.82 (0.72-0.93)	0.002	0.86(0.76-0.97)	0.016	0.007	0.95(0.91-0.99)
**85~100 (N=5170)**									
**MILK-EGG-SUGAR PATTERN**	reference								
**CARNIVOROUS PATTERN**	reference								
**HEALTHY PATTERN**	reference	0.89(0.82-0.96)	0.002	0.87(0.81-0.94)	0.001	0.83(0.76-0.90)	<0.001	<0.001	0.94(0.92-0.97)
**NORTHEASTERN PATTERN**	reference	0.91 (0.85-0.99)	0.025	0.89 (0.83-0.97)	0.006	0.91(0.84-0.99)	0.024	0.018	0.97(0.94-0.99)
**≥100 (N=2564)**									
**MILK-EGG-SUGAR PATTERN**	reference							0.039	1.04(1.01-1.08)
**CARNIVOROUS PATTERN**	reference								
**HEALTHY PATTERN**	reference					0.88(0.79-0.99)	0.041		
**NORTHEASTERN PATTERN**	reference			0.85 (0.76-0.95)	0.004	0.86(0.77-0.97)	0.016	0.003	0.94(0.91-0.98)

*Model= dietary pattern + gender + residence + educational level + marital status + smoke + alcohol assumption + exercise status + sleep + cognitive function + BMI + disease

** Q4 is the most adherent to that dietary pattern.
